# Same-Day Desensitization in Patients Who Experience Their First Reaction to a Platin Agent at the Oncology Day Unit: A Pilot Study to Safely Include This Technique Within the Multidisciplinary Pathways for the Diagnosis & Management of Hypersensitivity to Platin Agents

**DOI:** 10.3389/falgy.2022.868300

**Published:** 2022-06-16

**Authors:** Julián Borrás Cuartero, Roxana Farzanegan Miñano, María Cruz Torres Gorriz, Adrián Germán Sánchez, Raquel Cervera Aznar, Isabela Raducan, Jose Vicente Castelló Carrascosa, Alfredo Sanchez Hernandez, Ernesto Enrique

**Affiliations:** ^1^Allergy Department, Provincial University Consortium Hospital, Castellon, Spain; ^2^Allergy Department, Castellon University General Hospital, Castellon, Spain; ^3^Oncology Department, Provincial University Consortium Hospital, Castellon, Spain

**Keywords:** Same-Day Desensitization, Rapid Drug Desensitization, drug allergy, chemotherapy allergy, platin agents

## Abstract

One of the main objectives when assessing patients who react to antineoplastics must be to ensure that they receive the required treatments without delay. From January to July 2021, at the Allergy Department at the Provincial University Consortium Hospital a pilot study was performed in which those patients suspected of having suffered a type I hypersensitivity reaction (grade 1 or 2) following Brown's anaphylaxis severity grading to a platin agent at the Provincial University Consortium oncology day unit, and once the reaction was properly treated and completely resolved, were subjected to a new procedure named as Same-Day Desensitization, which consists in the reintroduction and administration of full chemotherapy dose by allergists on the same day of the reaction by following the 1 bag/10 step protocol, looking forwards to systematize same-day reexposure using Same-Day Desensitization, doing it in the safest way possible. In total, 9 oncological patients suspected of having suffered a type I hypersensitivity reaction (grade 1 or 2) to a platin agent received total dose administration the same day of the initial reaction by following Same-Day Desensitization 1 bag/10 step protocol, without presenting further reactions. The manuscript describes a new approach in the use of Rapid Drug Desensitizations in reactive oncologic patients in treatment with platin agents, presenting the first 9 cases of oncologic patients who have been submitted to this procedure.

## Introduction

Drug hypersensitivity reactions to chemotherapy (DHRsC) have been and currently still are an increasing worldwide problem which can lead to the premature discontinuation of first-line treatments, with oncologist then having to turn to second-line therapeutic options, therefore decreasing patients' quality of life and life expectancy. Fortunately, since the implementation of Rapid Drug Desensitization protocols (RDDs) by allergists, reactive patients are able to receive the recommended first-line therapy despite their DHRsC, thereby minimizing risks and maximizing patients life expectancy (1).

Chemotherapeutic schedules achieve maximum effectiveness when patients best adhere to the established therapeutic protocol. Of note, chemotherapy drugs have administration schemes that should not be altered because of the potential effect this can have on treatment efficacy (2). DHRsC may alter this fact, firstly by causing the interruption of first-line treatment administration in some cases, turning to second line therapeutic options, and secondly, by altering the chemotherapeutic administration rhythm with unforeseeable delays, thereby negatively affecting patients' quality of life, increasing the anguish associated with the whole process (3). Thus, appropriate management of the problem is now becoming essential.

Since the first publication, based on a series of 413 desensitizations using a 3-bag/12 steps protocol in 2008 emerged (4), allergists have gained new knowledge regarding chemotherapeutic allergy and RDDs:

1. Learning how to treat DHRsC during RDDs, being able to continue once the reaction is resolved, reaching total dose administration with no further complications (5).
2. There are precedents in the literature of same-day reexposure to chemotherapy after initial reaction in mild-to-moderate reactions, carried out by oncologists, without following a desensitization protocol, showing a higher rate of reactions in patients presenting features of IgE mediated reactions (6–8). Reexposure with a reduced infusion rate and additional premedication is usually successful in standard infusion reactions (SIRs) at grades I and II. However, reexposure should be avoided in patients who have recurrent SIRs despite premedication, those with grade III or higher SIRs, or suffering true type I reactions (9–11). Being the recommendation in these cases not to reexpose the patient to the causative agent until they have been evaluated by an allergy specialist.
3. A new classification for DHRsC based on phenotypes, endotypes, and biomarkers has been proposed. This approach encompasses classic Gell and Coombs classification of DHRs and reactions that do not fit this classification (12).
4. Patients suffering from a DHRsC during a drug provocation test (DPT) can receive total chemo dose the same day of the reaction by using de Restart Protocol described by Madrigal-Burgaleta et al. (13), who also nuances: “a phenomenon of temporary tolerance after the positive DPT reaction allows patients to safely receive the remaining treatment. If this fails, a one-bag desensitization may be attempted on the go” (14).
5. New protocols of RDDs have been described, using 1 bag protocol (15–17). A non-inferior approach respect to 3-bag protocols, which facilitates pharmacy's work and shortens time administration and total costs of all process.

The collaboration between oncologists and allergologists when assessing patients who suffer a first DHRsC to their antineoplastics must be to ensure that they receive the required treatments without delay and as safely as possible.

In the Provincial University Consortium Hospital in Castellon, thanks to the collaboration between the Allergy and Oncology Departments, allergists are usually alerted by the oncology day unit nurses when a DHRsC first occurs in the oncology day unit located in the hospital itself, allowing us to be present, asses and treat the reaction *in situ* and thereafter make the best therapeutic decision possible for each patient's context. This management also gives the opportunity to observe patients anguish experienced after suffering a DHRsC to their first-line therapy and be conscious of the loss of at least 1 therapeutic cycle (generally the one where the 1st reaction occurs), needing to wait until next chemo cycle, usually after allergological study is performed (done on time whenever possible to avoid further delays).

Given the above, and considering the global experience so far gathered in the academic literature regarding RDDs (4–19) the Allergy Department at the Provincial University Consortium Hospital questioned whether it would be possible the reintroduction and administration of full chemotherapy dose in patients suspected of having suffered an immediate type I hypersensitivity reaction (grade 1 or 2), following Brown's anaphylaxis severity grading (9), to a platin agent, on the same day of the reaction once the reaction was appropriately treated and resolved, doing it in the safest way possible, looking not to cause a mayor risk to the patient. If it were possible, unnecessary delays in the patient's therapeutic regimen could potentially be avoided.

To answer this question, the Allergy Department at the Provincial University Consortium Hospital considered the application of a new procedure named as Same-Day Desensitization, which consists in the reintroduction and administration of full chemotherapy dose, always done by allergists, in patients suspected of having suffered an immediate type I hypersensitivity reaction (grade 1 or 2) to a platin agent, on the same day of the reaction once the reaction was appropriately treated and resolved, taking advantage of the refractory period commonly referred to as post-anaphylaxis mast cell anergy (PAMA) or “empty mast cell (MC) syndrome” and by following the 1 bag/10 step protocol described by Sala-Cunill et al. (17), looking forwards to systematize same-day reexposure in these patients, using Same-Day Desensitization, doing it in the safest way possible, and always with an allergist being present, following a systematic pathway agreed in a multidisciplinary way and with staff trained to follow it safely.

## Materials and Methods

A pilot study was performed from January to July 2021, in which those patients suspected of having suffered an type I hypersensitivity reaction (grade 1 or 2) following Brown's anaphylaxis severity grading (9), to a platin agent at the Provincial University Consortium oncology day unit, once the reaction was properly treated and completely resolved, were subjected to Same-Day Desensitization taking as localization the same oncology day unit, meeting the security and quality criteria established by the RESCAL document of the Spanish Allergy and Clinical Immunology Society (SEAIC) (20) and the Risk and safety requirements for diagnostic and therapeutic procedures in allergology of the World Allergy Organization Statement (WAO) (21), being all process carried out by allergists in conjunction with the oncology day unit specialized nursing.

Up until now, in this study the implementation of Same-Day Desensitization has been avoided in two groups of patient: (1) In patients with suggestive symptoms of a type I hypersensitivity reaction: those who experience severe, life-threatening reactions (grade 3) (9); patients who did not need any further treatment with the suspected antineoplastic drug; and individuals with comorbidities where exposure to the drug in question might cause situations beyond medical control (such as in the case of the use of beta-blockers). (2) Those patients whose clinical scene was not suggestive of a type I hypersensitivity reaction.

For Same-Day Desensitization protocol, automatic high precision pumps, that allow the use of very small drug dilution administration rates were used. Starting at step 1 if the reaction had occurred from a few minutes after the infusion had started, or if the patient had additional comorbidities. Otherwise started at step 4 or further on when the infusion rate and treatment volume administered just before the reaction had been higher (Table 1), giving a flexible and personalized option for each patient depending on risk assessment, being this the first step of a wider assessment pathway.

**Table 1:** Example of the protocol used for the Same-day desensitization, using a non diluted 1 bag/10 step protocol, based on the protocol published by Sala-Cunill et al. (17).

**Example of dosing for 100 mg of oxaliplatin**.
•Concentrate solution for infusion contains:	5 mg/ml
•Total dose of oxaliplatin:	100 mg (20 ml)
•Total volume in the bag: 250 ml glucose solution + Oxaliplatin dose:	270 ml
•Normal concentration of the bag: 100 mg /270 ml:	0.37 mg/ml
•Example when the reaction appears at 40 ml of volume infused:	14.8 mg (40 ml)
•Remaining dose to be administered after the reaction:	85.2mg (230ml)
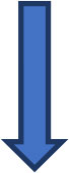	
Same-*day*desensitization (s-d-d) using the same baG (1 Bag/10 Steps)					
**STEP**	**Rate ml/hour**	**Time (min)**	**Administered volumen (ml)**	**Administered dose (mg)**	**Cumulative dose infused (mg)**
**1**	0.6	15	0.15	0.06	0.06
**2**	1.2	15	0.3	0.11	0.17
**3**	2.4	15	0.6	0.22	0.39
**4**	4.8	15	1.2	0.44	0.83
**5**	9.6	15	2.4	0.89	1.72
**6**	19.2	15	4.8	1.78	3.50
**7**	38.4	15	9.6	3.56	7.06
**8**	76.8	15	19.2	7.11	14.17
**9**	100	15	25	9.26	23.43
**10**	120	83.37	166.75	61.76	85.19
	Total (s-d-d)	218.4	230	85.20	85.20
	Previous to reaction:		40 ml	14.8 mg	
	Total (Administered)		270 ml	100 mg	

This was always done considering the Oncologist's opinion and with patient's informed consent. Approval from the Ethical Committee at our hospital was obtained before we started this work.

**Figure 1:**
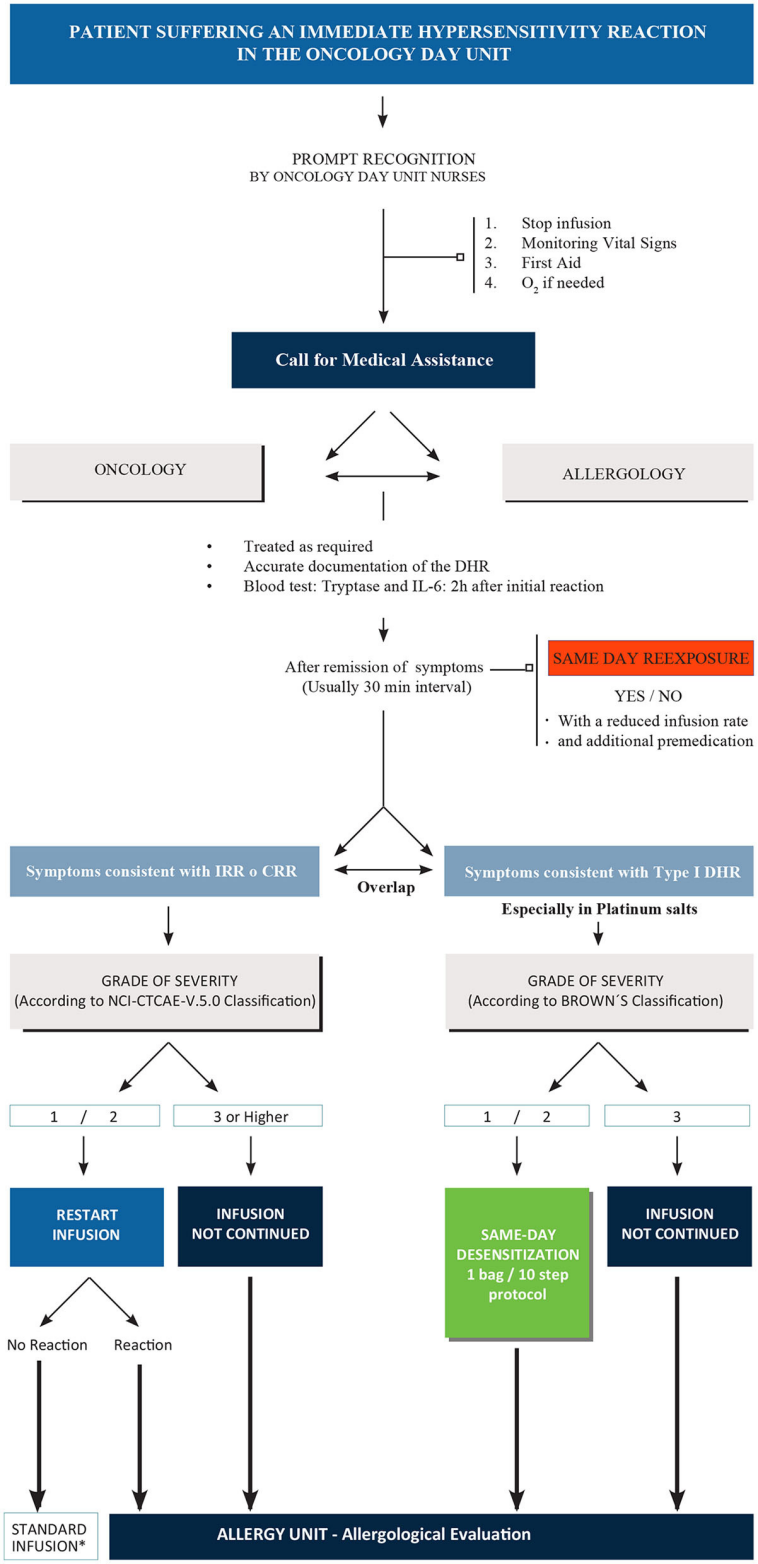
Complete diagnostic/therapeutic flowchart. IRR, infusion related reaction; CRR, cytokine release reaction; DHR, drug hypersensitivity reaction; NCI-CTCAE, National Cancer Institute Common Terminology Criteria for Adverse Events. * First-time patients controlled by allergist. For high risk patients, allergological evaluation is mandatory.

After Same-Day Desensitization, patients were all remitted for an allergological study, which was completed before their next chemotherapy cycle, being this key for the correct diagnosis and the subsequent tailored therapeutic plan. The complete flowchart carried out is shown in Figure 1.

## Results

In total, 9 oncological patients suspected of having suffered a type I hypersensitivity reaction (grade 1 or 2) to a platin agent (8 patients to oxaliplatin, 1 patient to carboplatin), received total dose administration the same day of the initial DHRsC, following Same-Day Desensitization 1 bag/10 step protocol, being all able to receive their complete chemotherapy dose without presenting further reactions (Table 2). Blood analyses were performed 2 h after their initial reaction to determine acute phase reaction serum tryptase and IL-6 levels. Furthermore, the patients were observed for 1 h after completing the Same-Day Desensitization.

**Table 2:** Clinically relevant data of the day of the initial DHRsC, for all 9 patients.

**Patient**	**1**	**2**	**3**	**4**	**5**	**6**	**7**	**8**	**9**
Drug involved N° treatment cicle Treatment/retreatment	Oxaliplatin 2° cicle Retreatment	Oxaliplatin 4° cicle 1° treatment	Oxaliplatin 4° cicle Retreatment	Oxaliplatin 8° cicle 1°treatment	Oxaliplatin 4° cicle Retreatment	Oxaliplatin 2° cicle Retreatment	Oxaliplatin 2° cicle Retreatment	Oxaliplatin 4° cicle Retreatment	Carboplatin 2° cicle Retreatment
Volume and rate of Infusion†	86 ml Normal infusion	235 ml Normal infusion	127 ml Normal infusion	40 ml Normal infusion	116 ml Normal infusion	146.8 ml Normal infusion	41.4 ml Normal infusion	150 ml Normal infusion	29 ml slow infusion
Simptoms during reaction	Acute generalized urticaria + pruritus	Acute generalized urticaria + pruritus	Mild acute urticarial + pruritus in low back	Itchy palms + abdominal pain	Acute generalized urticaria + pruritus	Acute generalized pruritus + mild urticaria	Itchy palms Mild exantema on the neck	Acute facial exantema + edema in auricular pavilions	Acute facial exantema, edema in auricular pavilions, cough + itchy palms
Suspected diagnosis and severity grade‡	Type I HR^§^ grade 1	Type I HR grade 1	Type I HR grade 1	Type I HR grade 1/2	Type I HR grade 1	Type I HR grade 1	Type I HR grade 1	Type I HR grade 1	Type I HR Grade 1/2
Same-day desensitization	Successfully completed	Successfully completed	Successfully completed	Successfully completed	Successfully completed	Successfully completed	Successfully completed	Successfully completed	Successfully Completed

*† Total volume infused of the culprit drug (ml) and rate of infusion just immediately before reaction started: Normal infusion corresponding to the stablished rate of infusion for each chemotherapeutic drug. ‡ Severity grade according to Browns Classification. **§** HR, Hypersensitivity reaction*.

Afterwards, all patients were remitted to the Allergy department for an allergological study to be performed (Table 3), being this one completed before their next chemotherapy cycle, which was subsequently administered without delay in the scheduled date. Next chemotherapy cycle was carried out along following a RDD or drug DPT, depending on the allergological study results (19): 8 of them continued with RDD, and the 9th patient was de-labeled after DPT (Table 3).

**Table 3:** Allergological study results and final diagnosis for the 9 patients.

**Patient**	**1**	**2**	**3**	**4**	**5**	**6**	**7**	**8**	**9**
Prick+ intradermal skin test performed ¶	Positive for oxaliplatin ID 1/1 20 × 14 mm + erythema + pruritus	Positive for oxaliplatin ID 1/10 8 × 10 mm + erythema + pruritus	Positive for oxaliplatin ID 1/100 15 × 9 mm + erythema + pruritus	Positive for oxaliplatin Prick 1/1 5 × 6 mm + erythema + pruritus	Negatives	Positive for oxaliplatin ID 1/10 10 × 10 mm + erythema + pruritus	Positive for oxaliplatin ID 1/100 11 × 12 mm + erythema + pruritus	Positive for oxaliplatin ID 1/100 10 × 13 mm + erythema + pruritus	Positive for Carboplatin ID 1/1 5 × 7 mm + erythema + pruritus
Tryptase Post reaction basal	13.5 μg/l 8.6 μg/l	30.9 μg/l 11.9 μg/l	8 μg/l 6.1 μg/l	10 μg/l 7.6 μg/l	4.5 μg/l -	4.5 μg/l -	6.9 μg/l -	4.5 μg/l -	4.4 μg/l -
IL-6 Post reaction basal	48.5 pg/ml 11.8pg/ml	1.2 pg/ml -	3.6 pg/ml -	1.8 pg/ml -	1.5 pg/ml -	2.3 pg/ml -	15.5 pg/ml -	5 pg/ml -	- -
Final diagnosis	Oxaliplatin IgE mediated allergy	Oxaliplatin IgE mediated allergy	Oxaliplatin IgE mediated allergy	Oxaliplatin IgE mediated allergy	Infusional reaction	Oxaliplatin IgE mediated allergy	Oxaliplatin IgE mediated allergy	Oxaliplatin IgE mediated allergy	Carboplatin IgE mediated allergy
No. of subsequent desensitization performed	11	3	1	2	DPT*	3	3	3	2

¶ Oxaliplatin dose: Prick test (1/1: 5mg/ml), Intradermal test (1/100: 0.05mg/ml), (1/10: 0.5mg/ml), (1/1: 5mg/ml). Carboplatin dose: Prick test (1/1: 10mg/ml), Intradermal test (1/100: 0.1 mg/ml), (1/10: 1mg/ml), (1/1: 10mg/ml). *DPT: Drug provocation test performed to oxaliplatin being well tolerated by the patient. Subsequent infusions carried on normally. **-:** Basal tryptase and IL-6 were not performed, taking into account that most values post reaction were considered low (normal) values, and due to the situation in our hospital's laboratory during Covid pandemic.

## Discussion

Chemotherapeutic schedules achieve maximum effectiveness when patients best adhere to the established therapeutic protocol. DHRsC may alter this fact, firstly by inducing forced changes to alternative treatments that might be less effective and secondly, by altering the chemotherapeutic administration rhythm with unforeseeable delays, thus, impairing patients prognosis and/or quality of life.

The first time an oncologic patient suffers a drug hypersensitivity reaction to their first-line treatment, the severity and nature of DHRsC and even the suspicious culprit drug involved in the reaction determines the physicians' decision to restart or not the treatment that same day.

In many settings, during a first reaction to chemotherapy, tendency in low-grade reactions is to reintroduce the drug, by slowing down infusion rates and increasing premedication. Reexposure with a reduced infusion rate and additional premedication is usually successful in mild to moderate SIRs at grades I and II allowing the patient to receive the full dose of the prescribed treatment on the same day of the reaction, therefore not losing that cycle. However, reexposure should not be attempted in patients who have recurrent SIRs despite premedication, those with grade III or higher SIRs, or suffering true type I reactions (9, 10). Specifically, patients with even mild symptoms of mast cell/basophil activation must be treated with caution, because reexposure to the causative agent could result in a fulminant and severe anaphylaxis, despite the premedication and reduced infusion rates that would have adequately managed most SIRs. In particular, these patients should not be considered candidates for additional premedication and an attempt at drug readministration using a slower rate of infusion (11), being the recommendation in these cases not to reexpose the patient to the causative agent until they have been evaluated by an allergy specialist with experience in drug allergy and desensitization, forcing therefore the suspension of that day's treatment, thus losing that treatment cycle.

In this report we present the first 9 patient cases suspected of having suffered a type I hypersensitivity reaction (grade 1 or 2) (9) to a platin agent who could receive their complete chemotherapy dose on the same day of the initial DHRsC, without presenting further reactions, by following a Same-Day Desensitization (1 bag/10 step protocol), and therefore avoiding unnecessary delays in the patient's therapeutic regimen.

The involvement and management of DHRsCs by allergists currently starts when patients are referred to the Allergy Department for an allergological study. These studies are key to designing tailored therapeutic plans which aim to de-label through DPTs, establish avoidance, or implement a RDD if required (11–14, 19). However, the presence of allergist during the initial reaction at the oncology day unit is practically scarce, and as shown in this study, allergists play an essential role when it comes to evaluating DHRsCs *in situ*, so we would like to emphasize how their accessibility is particularly important in these cases. Indeed, the roles they play in the initial evaluation and treatment DHRsCs can improve the global oncological therapeutic schedule designed for each patient. This further highlights the importance of multidisciplinary drug allergy teams in large hospitals, which should be led by expert allergists in close collaboration with oncologists, nurses, and pharmacists; likewise, expanding on this, access to drug allergy specialist centers (with trained staff and adequate installations) is a must.

This is an initial experience, nonetheless, more experience in this area is still needed to properly analyse the true safety of this procedure for these and other chemotherapy agents and to be able to consider its use in higher grade reactions in the future.

## Data Availability Statement

The original contributions presented in the study are included in the article/supplementary material, further inquiries can be directed to the corresponding author/s.

## Ethics Statement

The studies involving human participants were reviewed and approved by Comité Ético de Investigación con Medicamentos (CEIm) del Consorci Hospitalari Provincial de Castellón. The patients/participants provided their written informed consent to participate in this study.

## Author Contributions

JB and RF conceived the presented idea and developed the theory. MT, AG, and EE contributed to conception and design of the study. All authors contributed to the critical revision of the intellectual content, as to the final approval of the version to be published ensuring the integrity of the work.

## Funding

This work was supported by the Castellón Provincial Hospital Foundation.

## Conflict of Interest

The authors declare that the research was conducted in the absence of any commercial or financial relationships that could be construed as a potential conflict of interest.

## Publisher's Note

All claims expressed in this article are solely those of the authors and do not necessarily represent those of their affiliated organizations, or those of the publisher, the editors and the reviewers. Any product that may be evaluated in this article, or claim that may be made by its manufacturer, is not guaranteed or endorsed by the publisher.
